# Don’t Tell Us How Strong It Feels! Converging and Discriminant Validity of an Indirect Measure of Emotional Evidence Accumulation Efficiency

**DOI:** 10.3390/jintelligence14020019

**Published:** 2026-01-31

**Authors:** Rotem Berkovich, Deanna M. Barch, Nachshon Meiran, Erin K. Moran

**Affiliations:** 1 Department of Psychology, Ben Gurion University of the Negev, Beer-Sheva 84105, Israel; nachshon.meiran@gmail.com; 2 Department of Psychological & Brain Sciences, Washington University in St. Louis, St. Louis, MO 63130, USA; dbarch@wustl.edu (D.M.B.); ekmoran@wustl.edu (E.K.M.)

**Keywords:** emotion, evidence accumulation models, decision making, validity

## Abstract

The prevalent method for measuring emotional experiences is self-report scales. However, this method is prone to bias, affected by retrospective errors, and limited in studying individual differences due to variability in how individuals interpret scale values. In the present study, we tested the convergent validity of an alternative approach, which infers emotional components from computational modeling as applied to binary pleasant/unpleasant reports about affective images. Reaction times and choices were modeled to estimate the drift rate (efficiency of emotional evidence accumulation) and the boundary (decision caution). Participants (*N* = 191) also completed five self-report questionnaires assessing affect, anhedonia, depressive symptoms, and pleasure. Only one correlation reached evidence level (Bayes Factor > 10): Higher consummatory pleasure was negatively associated with drift rate for unpleasant emotions (*r*(178) = −0.258). This suggests that individuals who typically experience greater in-the-moment pleasure accumulate evidence less efficiently toward unpleasant judgments. Other correlations were absent or inconclusive, potentially reflecting differences in temporal focus and in the specific facets of emotion for each measure. Overall, these results provide some initial support for the convergent and discriminant validity of the drift rate as an indirect measure of online emotional experience.

## 1. Introduction

Measuring an individual’s emotional experience has long presented significant challenges. Currently, the predominant method for measuring emotion involves self-reported scales. However, using self-report scales to measure emotions has many limitations (see Mauss & Robinson, 2009). For example, retrospective emotional reports are often influenced by stereotypical information, leading individuals to report emotions that align with gender stereotypes (Fischer et al., 2004). Moreover, participants may underestimate the intensity of their emotions when reflecting on past experiences (Kaplan et al., 2016), and their reports might be biased by their current appraisals of events (Levine et al., 2001). Some of these issues, such as retrospective bias, can be resolved by using momentary self-reports. However, even real-time self-reports are not immune to biases. A notable example is the initial elevation phenomenon, where participants tend to provide higher negative ratings during the first assessment (Anvari et al., 2023). Furthermore, individual differences in the ability or willingness to report emotion add another layer of complexity. One example is alexithymia. Individuals high in alexithymia often struggle to articulate their emotional experiences (Lane et al., 1997). Beyond these issues, self-report scales are inherently problematic because we cannot guarantee that participants’ ratings are on the same scale. For example, two individuals may rate an emotion as “2—very unpleasant,” but their subjective experiences could differ, with one person feeling significantly more unpleasant than the other (Bartoshuk et al., 2005). Finally, raw self-reports offer little insight into the generative process behind the rating. For example, apparent individual differences may reflect differences in decision policies but not in felt intensity. These limitations highlight the potential challenges of heavy reliance on self-report scales to measure emotional experience.

Due to the numerous limitations associated with self-report rating scales to measure emotional experience, a complementary approach has been proposed: utilizing Evidence Accumulation Models (EAMs) to measure emotional experiences (Givon et al., 2020). EAMs are a family of models grounded in the principle that decision-making involves the accumulation of evidence from a noisy environment until sufficient evidence has been accumulated and a threshold has been reached (Ratcliff & Smith, 2004). The basic notion is that reporting about having an emotional experience is a decision, and this decision must rely on some (including inner) information.

**Figure 1:**
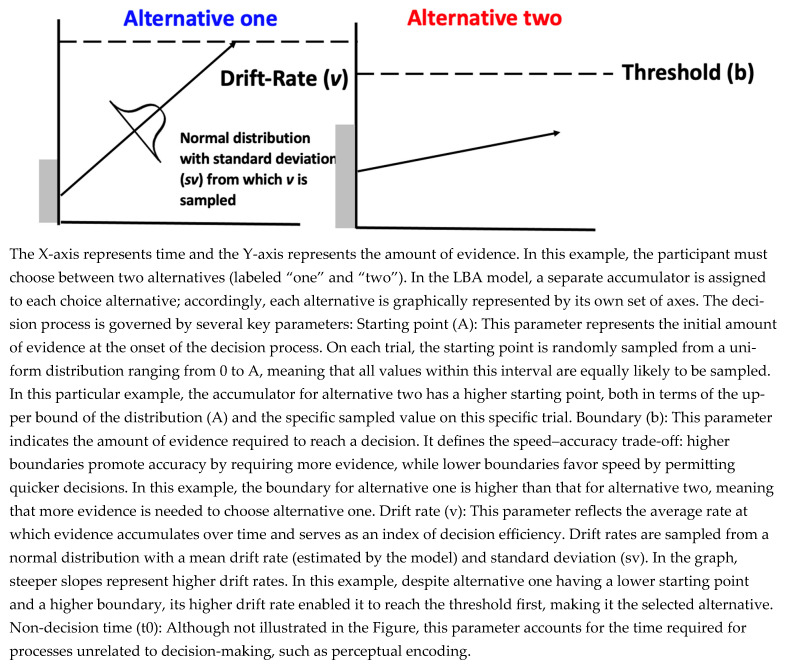
A graphical representation of the LBA model illustrating a single trial.

In EAM, the decision-making process is modeled by assuming that evidence is accumulated in accumulators, whose behavior is characterized by several key parameters: the mean rate of evidence accumulation, the noise in the evidence accumulation process, the threshold of evidence required for decision-making, the initial evidence present in an accumulator before the decision process begins, and the duration of non-decision processes (e.g., motor preparation). In the current investigation, we focus on a specific EAM: the Linear Ballistic Accumulation model (LBA; Brown & Heathcote, 2008), as this model was used in earlier work that has established EAM as a tool to model emotional experiences (Berkovich & Meiran, 2023; Givon et al., 2020, 2022, 2023). Therefore, we chose this model to align with previous work. See Figure 1 for a graphical representation of the LBA model.

The LBA modeling approach to measure emotional experience involves a task in which participants are presented with emotion-evoking images and are asked to indicate whether the image makes them feel pleasant or unpleasant. That is, both the proposed method and the present work focus on emotionally aware experience as it is evoked online. While the task in this method involves self-reporting emotion, it is not employed in its traditional manner. Instead, participants’ pleasant/unpleasant responses and reaction times for each image are integrated into an LBA model, which generates latent variables that capture the decision-making process. In contrast to traditional self-report scales, where emotional experience components (e.g., efficiency of emotional evidence accumulation) are directly derived from participants’ subjective ratings, the LBA approach infers those emotional experience components indirectly from the model parameters, thereby reducing susceptibility to self-report bias, and, in addition, providing insight into the process that produces the reports, such as whether observed differences reflect sensitivity to affective information or differences in the decision caution. Moreover, by employing a specific model estimation technique (Berkovich & Meiran, 2024), this approach enables the comparison of emotional components across individuals by ensuring that participants’ parameters are all on the same scale. Specifically, since LBA’s model parameters are scale-free, it is necessary to generate a scale, which is often performed by fixing one parameter for all individuals. For example, LBA’s sv is often fixed at one to establish a scale, meaning that all the other parameters, such as mean drift rate, are expressed in terms of this scale. This approach, however, implicitly assumes that there are no individual differences in the fixed parameter, an assumption that may be violated and can compromise the estimation of the remaining model parameters. To address this issue, we set the scale at the population level rather than at the individual level. Additionally, using this method, and given that the drift rate parameter is on a ratio scale (Ratcliff, 2014), the new method makes it possible to conclude, for instance, that one participant’s mean drift rate, and by extension, emotional intensity, is twice as large as that of another individual.

Previous research using the LBA modeling approach to measure emotions has demonstrated that the rate of evidence accumulation increases monotonically with the mean (un)pleasantness rating (Berkovich & Meiran, 2023), suggesting that the rate of evidence accumulation may serve as a measure of the intensity of the emotional experience. These findings are particularly noteworthy because participants make dichotomous decisions, meaning that the intensity of their emotions is not directly reported but inferred through the model. It was further demonstrated that the drift rate for negative emotional experiences was higher for women than for men, but only for unpleasant experiences (Givon et al., 2023). This finding suggests that the drift rate in emotion report tasks does not merely reflect general task efficiency, since it reveals gender differences that are valence-specific. Another key finding pertains to the boundary parameter. It has been shown that the boundary is sensitive to speed versus accuracy emphasis (Givon et al., 2022): in pleasant/unpleasant decisions, an emphasis on accuracy increases the boundary, while an emphasis on speed decreases it. Thus, the LBA’s latent parameters appear to capture fundamental components of emotional experiences.

These findings provide preliminary evidence regarding convergent validity, the extent to which two measures capture the same underlying construct. Specifically, in this case, convergent validity concerns the drift rate and boundary parameters in LBA regarding emotion reports. For instance, the observed monotonic relationship between valence ratings and drift rate suggests that the drift rate reflects the intensity of emotional experiences. Although those prior findings suggest that drift rate can measure emotional intensity, these findings are at the normative (group) level, and not at the level of individual differences, which is what we are focusing on in the present study. Since evidence at these two levels does not always align, we avoid adopting this terminology here and use the term emotional evidence efficiency rather than emotional intensity. Additionally, the available evidence is insufficient to fully capture the scope of what these model parameters represent. Consequently, the aim of this study was to evaluate the validity of these two LBA parameters in the context of binary un-/pleasantness reports. Specifically, we examined convergent validity by testing whether these parameters relate to measures that are theoretically proximal to stimulus-elicited emotional experience and discriminant validity by assessing their associations with indexing measures that are conceptually distinct.

To address this aim, participants engaged in the pleasant/unpleasant experience task (i.e., “the emotion task”), during which they were presented with emotion-evoking images sourced from The Nencki Affective Picture System (NAPS; Marchewka et al., 2014). The selected images were characterized by moderate emotional intensity. Participants’ reports and reaction times were integrated into an LBA model. The primary LBA parameter of interest is the drift rate, which reflects the mean (across trials) rate of evidence accumulation. The drift rate can be modeled separately for accumulators corresponding to correct and incorrect responses. Within the context of the pleasant/unpleasant task, accuracy was determined relative to normative ratings; correct responses corresponded to normative choices, while incorrect responses reflected counter-normative choices. This classification is enabled by prior findings demonstrating that (in)accuracy effects observed in perceptual decision-making tasks also occur in emotion reports, where participants’ accuracy is assessed relative to normative responses (Givon et al., 2022).

**Figure 2:**
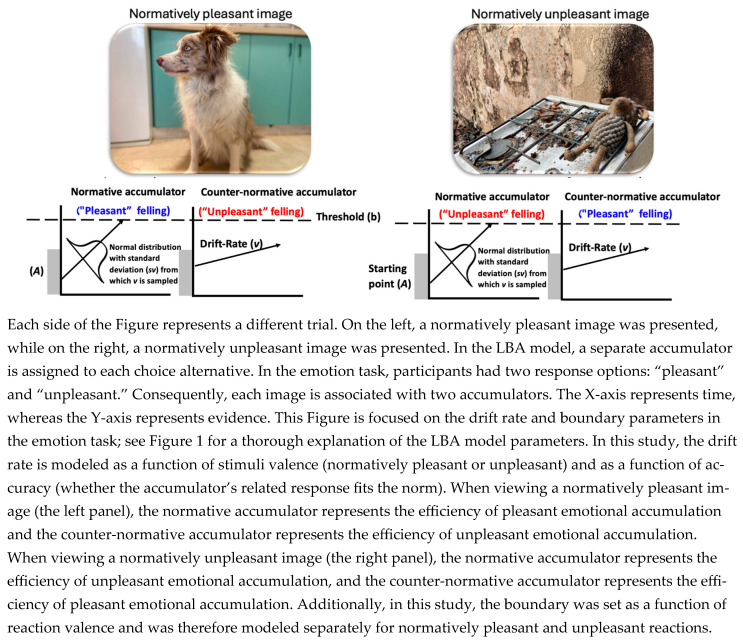
Graphical representation of the LBA model in the emotion task, illustrating two trials.

By considering both accuracy and the normative nature of the stimuli (pleasant vs. unpleasant norms), four distinct drift rate parameters were estimated: (1) normative accumulator for normatively pleasant stimuli; (2) normative accumulator for normatively unpleasant stimuli; (3) counter-normative accumulator for normatively pleasant stimuli; and (4) counter-normative accumulator for normatively unpleasant stimuli. These parameters can be conceptualized as representing two distinct emotional states: efficiency of pleasant and unpleasant emotional evidence accumulation. The efficiency of pleasant emotional evidence accumulation is associated with the first and fourth parameters. In the first parameter, the efficiency of emotional feeling accumulation aligns with the normative expectation, where the norm corresponds to a pleasant experience. Similarly, the fourth parameter corresponds to a feeling that is counter-normative to an unpleasant feeling, thereby also indicating the efficiency of pleasant emotional accumulation. A parallel logic applies to the second and third parameters, which are associated with the efficiency of unpleasant accumulation: the second reflects alignment with a normative unpleasant response, while the third represents a counter-normative response to a pleasant stimulus, both contributing to an efficiency of unpleasant emotional accumulation. The second LBA parameter of interest is the boundary, which represents the amount of evidence required to reach a decision. In the pre-registration, the boundary parameter was specified as varying as a function of stimulus valence, yielding separate boundaries for normatively pleasant and normatively unpleasant stimuli. In retrospect, we realized that this specification was theoretically imprecise. Allowing the boundary to vary as a function of stimulus valence implicitly assumes that participants set their decision threshold after knowing whether a stimulus is pleasant or unpleasant. This assumption is difficult to justify within evidence accumulation frameworks, as decision thresholds are set prior to evidence accumulation. We therefore refit the model so that the boundary varied as a function of the response (pleasant versus not pleasant), capturing how much evidence an individual needs to accumulate to each type of response. Notably, the correlation reported in the main text was also observed under the pre-registered model. However, the pre-registered specification yielded an additional correlation that did not replicate under the theoretically coherent model, suggesting that the initial specification may have inflated the apparent evidence for convergent validity. Full details of this alternative model and its results are provided in the Supplementary Materials. Accordingly, the final model includes two boundary parameters corresponding to pleasant and unpleasant responses. Those parameters are graphically explained in Figure 2.

In addition to the pleasant/unpleasant task, participants completed five self-report questionnaires: The Positive Affect Negative Affect Schedule (PANAS; Watson et al., 1988) has been widely used to measure an individual’s experience of positive and negative affects across different time scales. We administered 10 positive affect items (interested, excited, strong, enthusiastic, proud, alert, inspired, determined, attentive, and active) and 10 negative affect items (distressed, upset, guilty, scared, hostile, irritable, ashamed, nervous, jittery, and afraid), asking for participants’ current experience of these emotions. Participants were instructed to rate the extent to which they were currently experiencing each emotion, using a scale ranging from 1 (very slightly or not at all) to 5 (extremely). This questionnaire results in two scales: current positive emotional experience and current negative emotional experience. Both the positive and negative affect scores have good internal consistency and strong convergent and divergent validity (Watson et al., 1988). The Mood and Anxiety Symptoms Questionnaire (Buckby et al., 2007; Clark & Watson, 1991) broadly assesses the severity of various aspects of anxiety and depression over the past week. We administered a subset of 38 items (not including the suicide item), with each item describing a specific feeling, sensation, or experience (e.g., “Startled easily” and “Felt hopeful about the future”). Participants were asked to evaluate the extent to which they have experienced each statement over the past week, including the current day, using a scale ranging from A (Not at all) to E (Extremely). The MASQ generates two scales: anxious arousal and anhedonic depression. Each of these subscales has good internal consistency and construct validity (Xiao et al., 2015). The Temporal Experience of Pleasure Scale (TEPS; Gard et al., 2006) is an 18-item scale, with each item reflecting a specific feeling or experience (e.g., “The smell of freshly cut grass is enjoyable to me”), that assesses self-reports of both consummatory pleasure and anticipatory pleasure in general (not a specific time frame). Participants were instructed to evaluate the extent to which each statement generally applies to them, using a scale ranging from 1 (Very false for me) to 6 (Very true for me). If participants have never encountered the specific experience described in an item, they are directed to consider a similar experience they have had. The two subscales have reasonable internal consistency, though the evidence for the separability of consummatory and anticipatory pleasure is mixed (Garfield et al., 2016; Ho et al., 2015; Li et al., 2018). The Center for Epidemiologic Studies Depression Scale—Revised (CESD-R 10; Andresen et al., 1994) is a 10-item self-report scale assessing the severity of symptoms of depression. Participants were instructed to indicate how often they had experienced the given symptom over the past week using a scale from rarely to all of the time. The scale has strong validity (Van Dam & Earleywine, 2011). The Snaith–Hamilton Pleasure Scale (SHAPS; Snaith et al., 1995) is a 14-item measure assessing an individual’s self-reported pleasure in response to a range of experiences over the last few days (e.g., “I would get pleasure from helping others”). Participants were instructed to rate each statement on a scale ranging from 1 (Strongly disagree) to 4 (Strongly agree). It has good internal consistency and construct validity (Langvik & Borgen Austad, 2019; Montoro et al., 2022). Those questionnaires yielded eight scales: current positive emotional experience (PANAS), current negative emotional experience (PANAS), anxiety (MASQ), anhedonia (MASQ), anticipatory pleasure (TEPS), consummatory pleasure (TEPS), severity of depressive symptoms (CESD-R 10), and (un)pleasure scale (anhedonia, SHAPS). Correlations between these self-report scales and the drift rate and boundary parameters were analyzed. After submitting our pre-registration and engaging more deeply with the literature, we came to realize that certain pre-registered hypotheses lacked a robust theoretical foundation. Therefore, we will focus here on discussing hypotheses supported by an established theoretical framework (to be clear, a number of these were not supported). Additionally, several correlations were examined as part of pre-registered exploratory analyses, not driven by specific hypotheses.

### Hypotheses

The pre-registration included hypotheses concerning both drift rate and boundary parameters. However, the boundary-related hypotheses were motivated by findings from work that has not yet been published. Therefore, in accordance with the journal’s policy, those hypotheses are not detailed here. These hypotheses are documented in the pre-registration and were not empirically supported. Accordingly, all hypotheses detailed below pertain exclusively to the drift rate parameters. Additionally, all hypotheses were formulated with the intention of testing convergent validity. However, upon closer consideration, we retrospectively recognized that some of these hypotheses, in fact, bear more directly on discriminant validity, given the conceptual distance between the real-time stimulus-elicited emotional experience measured with our method as compared with retrospective and trait-like reports characterizing most of the self-report measures being examined. Accordingly, the manuscript now frames the results as bearing on both convergent and discriminant validity. Yet, this reframing is post hoc and should be interpreted as such. Since this distinction between measures relevant to convergent and discriminant validity was determined post hoc, we do not elaborate on it here. The rationale and classification are described in detail in the Discussion.

It has been found that in tasks where participants are instructed to report their current emotional experience that is being evoked by a specific stimulus, the drift rate increases monotonically with valence ratings (Berkovich & Meiran, 2023). Thus, we assume that the drift rate parameters represent the intensity of the feeling that is being experienced. Therefore, with respect to the PANAS scales, we predicted that the drift rate parameters for pleasant emotional accumulation efficiency would be positively related to the current positive emotional experience scale and negatively related to the current negative emotional experience scale. Using the same logic, we predicted that the drift rate parameters for unpleasant emotional accumulation efficiency would be positively related to the current negative emotional experience scale and negatively related to the current positive emotional experience scale. This hypothesis assumes that people who report having more current (un)pleasant feelings would also evaluate a new stimulus as more (un)pleasant.

An additional hypothesis pertains to the MASQ Anhedonia scale. Individuals with elevated levels of anhedonia are characterized by a reduced ability to experience pleasure (Rømer Thomsen et al., 2015). Consequently, we hypothesized that participants with higher levels of anhedonia would demonstrate increased drift rate parameters for unpleasant emotional accumulation efficiency and decreased drift rate parameters for pleasant emotional accumulation efficiency. Given that the SHAPS questionnaire also assesses anhedonia, we formulated the same hypotheses for this measure^1^.

The final hypothesis focuses on the TEPS Consummatory Pleasure scale, which evaluates the degree of pleasure derived from “current” imaginable specific situations (“The smell of freshly cut grass is enjoyable to me”). This contrasts with the TEPS Anticipatory Pleasure scale, which measures the anticipated pleasure associated with such situations. Given that the Consummatory Pleasure scale specifically assesses pleasant emotional experiences, we hypothesize a positive correlation between this scale and the drift rate parameters associated with pleasant emotional accumulation efficiency, and a negative correlation with the drift rate parameters associated with unpleasant emotional accumulation efficiency.

A comprehensive summary of all hypotheses and exploratory analyses is provided in Table 1.

## 2. Materials and Methods

The experiment was pre-registered here (https://osf.io/93qje/overview, accessed on 11 January 2026).

### 2.1. Sample Size Determination

Given the absence of established power analyses specific to the type of analyses we conducted (using EAM to derive latent variables and subsequently testing their correlations with other variables), we adopted an approximation approach. Specifically, in the absence of established power analysis procedures for LBA model estimation, power was approximated based on the planned correlation analyses. We conducted a power analysis using the G*Power software (version 3.1). The test was specified as a *t*-test for a linear bivariate regression with one group and a slope effect size, utilizing a two-tailed test. The alternative hypothesis (H1) assumed a slope of 0.25, while the null hypothesis (H0) posited a slope of 0. The analysis was conducted with a desired power of 0.80, and a standard deviation for both x and y was set to the default and assumed to be 1. To account for multiple comparisons, the Bonferroni correction was applied with an initial alpha of 0.05, which was divided by 7, yielding a corrected alpha of 0.00714. This value was subsequently rounded to 0.0073 for a slightly more conservative threshold. The analysis indicated that a sample size of 190 participants would be required.

### 2.2. Participants

Participants in this study were members of the Washington University, in the St. Louis community. Participants were recruited via Sona Systems, which is an online subject recruitment platform. All participants were above the age of 18 and native English speakers. After the initial exclusion based on completion (before looking at the data), we had a total of 191 participants. Our target sample was *N* = 190. However, due to the recruitment process, in which multiple participants were invited to take part simultaneously, the final number of collected datasets slightly exceeded this target, as the exact number of participants completing the task could not be determined in advance. Prior to applying data exclusion criteria based on participants’ performance, a total of 191 participants were included in the dataset, of whom 135 were females (mean age = 20.21) and 56 were males (mean age = 20.45). The participants were part of a larger study investigating motivation and reward processing. The Washington University Institutional Review Board approved the protocol used in this study and all participants provided informed written consent. Participants completed a 1.5 h online visit via Zoom. Following consent, participants completed a demographic questionnaire and self-report questionnaires (described below) via Redcap. Following the online session, participants were sent a link to complete the PANAS and behavioral task on their own time following this session. They were paid USD 30 for completion of the online Zoom session and were given a bonus for completion of all the study procedures, up to an additional USD 20. Participants completed the study across a ~3-day period, in two or three separate sessions, and were paid upon completion of the study.

### 2.3. Task Description

The study consisted of two parts: the questionnaires and the behavioral task. The questionnaires are fully described in the Introduction. The behavioral task is described in detail below.

The behavioral task is based on two tasks, the emotion task and the letter tasks, with trials of both tasks interleaved:

The emotion task: Participants were presented with emotion-evoking images taken from the NAPS database (Marchewka et al., 2014) and were instructed to report whether the picture made them feel pleasant or unpleasant. This binary report was made by pressing the ‘p’ key for pleasant reports and the ‘z’ key for unpleasant reports. After each response, a feedback frame appeared around the image: a green frame for pleasant reports and a red frame for unpleasant reports. All the images had established norms and were categorized into one of five categories: animals, objects, landscapes, faces, and people. Image selection was based on both the category and the normative ratings. In the practice phase, 15 images were presented, distributed across the following categories: three from the animal and object categories, four from the landscape category, and five from the face category. Of these images, seven were normatively pleasant (mean valence = 6.94) and eight were normatively unpleasant (mean valence = 3.91), using a scale of 1–9, where 1 represents very unpleasant, 9 represents very pleasant, and 5 represents neutral valence. In the main experimental blocks, thirty images were used from each category, with half of the images in each category being normatively pleasant and half normatively unpleasant. However, in the animal category, due to dataset limitations, 17 images were normatively pleasant and 13 were normatively unpleasant. In the experimental blocks, normatively unpleasant images had a valence range from 2.42 to 4.36 (mean valence = 3.83), while the normative ratings of the pleasant images ranged from 5.64 to 6.84 (mean valence = 6.27).

The letters task: In this task, participants were presented with one of two letters, either “x” or “o,” and their task was to identify which letter was displayed. This binary response was made by pressing the corresponding key (i.e., the ‘x’ key for “x” and the ‘o’ key for “o”). Participants were informed that the objective of the task was to assess whether their emotional state influenced their performance on the letter identification task, while the true purpose of the task was to minimize demand characteristics.

**Figure 3:**
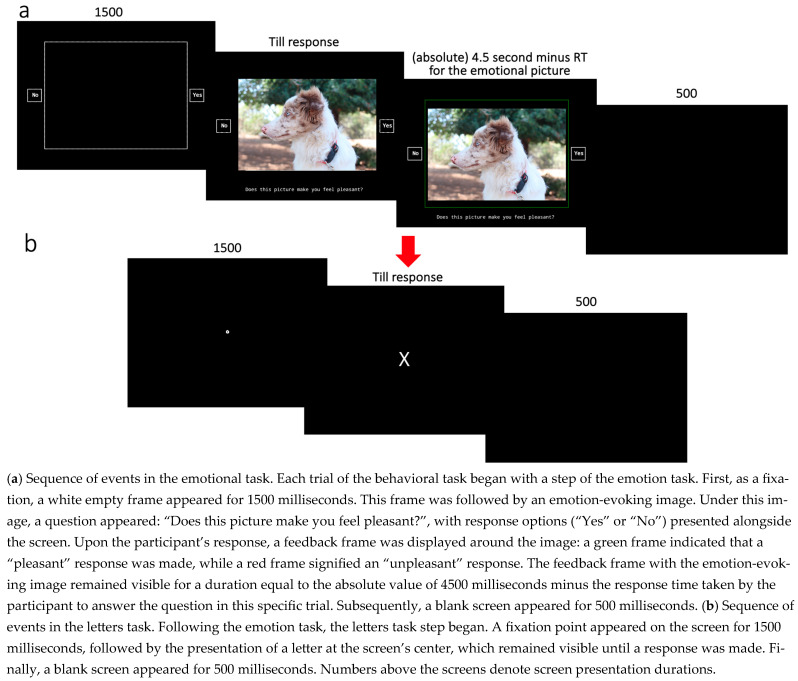
Sequence of events in a trial.

In both tasks, participants’ responses and reaction times were recorded. To minimize potential confusion or forgetfulness regarding the correct keys for the emotion task, since this was the focus of the study, we employed two strategies. The first strategy involved displaying the possible response options alongside the screen during each trial of the emotion task. The second strategy utilized reaction times: if participants pressed an irrelevant key (e.g., pressing ‘o’ instead of ‘p’ in the emotion task), the feedback frame would not appear, allowing them to correct their response. However, the initial reaction time was still used in subsequent analyses. Additionally, the final response considered was the second (task-appropriate) response. An example trial sequence of both tasks is illustrated in Figure 3.

### 2.4. Procedure

The study was conducted online. Prior to participation, participants provided informed consent. Then, participants were asked to complete a demographic form along with self-report questionnaires (described above). All data were collected and stored in Redcap. Following the online study session, participants were sent a link to complete both the PANAS and the behavioral task on their own time. Participants received one link per day for the next three days. Participants completed the PANAS each day prior to completing the behavioral task.

In the behavioral task, the initial block consisted of a training session for the letters task, comprising 10 trials. This block also included feedback represented by a green ‘thumb up’ symbol for correct answers and a red ‘thumb down’ symbol for incorrect answers. The second block encompassed a training session for the emotion task, comprising 3 trials. This was followed by the third block, which served as a training session for the keys for both tasks. In this block, participants were exposed to each of the four possible responses (“x”, “y”, “yes”, and “no”) in separate trials and were instructed to press the key corresponding to each response. This block included 16 trials in which each trial was followed by feedback provided after each trial using the same symbols as in the first block. The final training block involved the emotion and letters tasks together, just as in the experimental blocks. This block consisted of 6 trials, with each trial containing both tasks. After the training blocks, participants progressed to 3 similar experimental blocks, each comprising 16 to 17 trials. In the second and third sessions, the main experimental blocks were built in the same manner as in the first session, but with only one short practice block: the emotion and letters tasks together for 2–3 trials in each session. Each session contained 50 unique emotion-evoking pictures.

### 2.5. Data Exclusion

We used a separate data exclusion plan for the emotion task and the questionnaires. For the emotion task, data exclusion was implemented according to the following pipeline: Participants were excluded if a technical or equipment failure resulted in the loss of more than 50% of their data, if they had not completed at least two parts of the experiment, or if they failed to complete the experiment in the correct sequence with reasonable time intervals between parts (participants who completed different parts of the task more than one week apart were not included in the analysis). Participants who completed part 3 before part 2 were retained only if they had completed part 1 first. Following these exclusion criteria, the target sample size was *N* = 190. Stimuli were excluded if they had less than 60% agreement among participants regarding their valence. Additionally, participants with normative response rates of more than 2.5 standard deviations below the mean of the entire sample were excluded. Trials were excluded if the reaction times were greater than 7 s or shorter than 200 milliseconds, as well as trials with reaction times more than 3.5 standard deviations above the mean reaction time (as determined per condition within subject). For the questionnaire data, a different approach was employed: We plotted the data for each questionnaire in order to visually inspect outliers^2^.

### 2.6. Modeling and Analysis Plan

In this study, we aimed to examine the correlations between the LBA model parameters (derived from modeling the emotion task) and the questionnaire data, quantifying the strength of evidence using Bayes Factors. Therefore, we provide a detailed explanation of how the LBA model parameters and questionnaire scores were obtained for each participant.

The emotion task: To estimate the model parameters for the emotion task, we utilized the ‘ggdmc’ package (version 0.2.6.0; Lin & Strickland, 2020), which enables Bayesian hierarchical estimation of the LBA model. This method provides individual-level parameter estimates while imposing the constraint that all participants belong to the same underlying population. The estimation process employs a Markov Chain Monte Carlo (MCMC) algorithm and consists of two main steps: The first is a burn-in period in which multiple chains explore the parameter space to identify a high-probability region. The second is the actual search, in which the chains focus their search within the high-probability region identified during the burn-in period. This search provides the most probable parameter estimates for each participant.

To implement this procedure, distribution priors must be specified for each parameter at both the individual and the population levels. Additionally, the number of chains and the number of samples per chain must be defined. We estimated two LBA models: the Null model and the Main model. In the NULL model, drift rates were estimated separately for normative (“correct”) and counter-normative (“incorrect”) responses. Mean priors for this model were derived from the posterior NULL reliability internal consistency model, as reported in a yet unpublished paper, while the population sv set the scaling (Mu-sv = 1), ensuring a uniform measurement scale across all participants (Berkovich & Meiran, 2024). More specifically, those mean priors were as follows: A = 1.967, b = 1.516, t0 = 0.193, v normative = 2.433, v counter-normative = 0.663, and sv = 0.998. In the Main model, the drift rate was estimated as a function of both response “accuracy” (normative vs. counter-normative responses) and stimulus valence (pleasant vs. unpleasant), and the boundary parameter was set as a function of response-valence. All other model parameters were estimated across conditions. The posterior estimates from the Null model were used as mean priors for the Main model. In both the Null and the Main model, the scale priors were as follows: A = 0.6, b = 0.6, t0 = 0.05, sv = 0.04, v normative = 0.8 (for both (un)pleasant categories), and v counter-normative = 0.08 (for both (un)pleasant categories). In both models, the default number of chains (three times the number of model parameters) was used. During the burn-in period, each chain sampled 1000 samples, and the actual search involved 12,000 samples. Additionally, in the Main model, thinning was set to 12, meaning that every 12th sample was retained to improve memory efficiency and to reduce autocorrelation between successive samples.

To evaluate chain convergence, we utilized the potential scale reduction factor (PSRF), which assesses convergence by comparing within-chain variance to between-chain variance. Chains are considered to have converged adequately when the PSRF value is below 1.1 (Brooks & Gelman, 1998). Following this assessment, we compared the two models to determine whether the Main model provides a better fit to the data than the Null model. To achieve this, we computed the Deviance Information Criterion (DIC; Spiegelhalter et al., 2014) for each participant and summed these values separately for each model. We then compared the aggregated DIC values across models. Additionally, we examined the number of participants for whom the Main model provides a superior fit. Together, these two pieces of information informed our decision regarding the model that best fits the data. Additionally, we decided to include a model fit assessment that was not pre-registered by evaluating each model’s Root Mean Square Error of Approximation (RMSEA; Steiger & Lind, 1980). In the context of the EAM’s, an RMSEA value below 0.08 is typically interpreted as indicating a good model fit (Schubert et al., 2017).

The questionnaires: For each questionnaire, raw response data was transformed into a single or dual scale for each participant. Scale scores were calculated by summing the relevant items, with reverse-scored items appropriately adjusted. Additionally, for the PANAS, since the study was conducted across 2–3 sessions and PANAS was administered in each session, the final PANAS score for each participant was computed as the mean score across sessions.

As previously noted, we examined the correlation between questionnaire scores and LBA model parameters’ mean posteriors. Given the multiple comparisons being conducted, a more stringent Bayes Factor (BF) threshold was applied than the commonly used criterion. To support H1, we adopted a BF threshold greater than 10, and to support H0, we used a BF threshold less than 1/10.

## 3. Results

Prior to data analysis, we implemented our data exclusion pipeline. For the emotional task, this process led to the exclusion of 35 images and six participants. Additionally, a total of 367 trials were excluded based on the following criteria: 142 trials with response times (RTs) exceeding 7 s, 12 trials with RTs shorter than 200 milliseconds, and 213 trials with RTs more than 3.5 standard deviations above the mean RT (determined per condition within subject).

For the questionnaire data, we plotted each questionnaire and conducted a visual inspection to identify outliers; however, no outliers were detected. The plots that were used to inspect those outliers can be found in the Supplementary Materials. It is important to note that although data from 185 participants were included in the LBA model (from an initial sample of *N* = 191, with six participants excluded), not all of these participants completed the questionnaires. As a result, the final correlation analyses were conducted on a sample of 167–184 participants. Detailed information on the number of respondents per questionnaire, as well as descriptive statistics (means and standard deviations), is presented in Table 2.

For both the Null and Main models, the PSRF values for all participants were below 1.1, with the highest value being 1.0011, indicating satisfactory chain convergence. The summed DIC values for each model were 38,743.09 for the Null model and 36,246.18 for the Main model. Given that a lower DIC value reflects a better model fit, this result provides decisive evidence in favor of the Main model over the Null model. Furthermore, for 64.32% of the sample, the DIC was lower in the Main model compared to the Null model. Collectively, these findings support the Main model, suggesting that the drift rate parameters vary as a function of valence and that the boundary varies as a function of reaction. Additionally, for both models, RMSEA values fell below 0.08, indicating a good model fit (RMSEA = 0.0066, 95% CI [0.006, 0.007], RMSEA = 0.0477, and 95% CI [0.0472, 0.0483], respectively). In the Null model, the mean posteriors were as follows: A = 1.501, b = 1.388, t0 = 0.205, v normative = 2.138, v counter-normative = 0.44, and sv = 0.967. Those mean posteriors were also used as mean priors for the Main model. In the Main model, the mean posteriors were as follows: A = 1.345, b positive valence = 1.357, b negative valence = 1.55, t0 = 0.221, v normative positive valence = 1.985, v normative negative valence = 2.21, v counter-normative positive valence = 0.587, v counter-normative negative valence = 0.291, and sv = 0.952.

**Figure 4:**
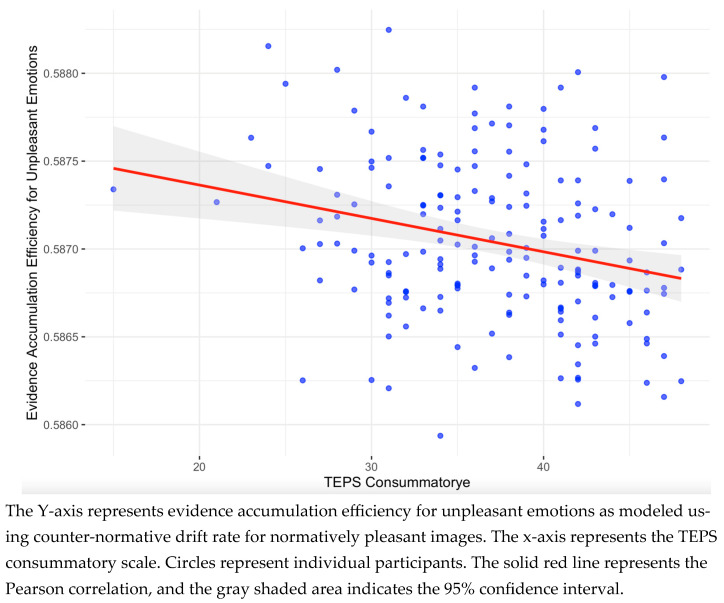
Scatterplot of the notable correlation between the drift rate parameter and questionnaire scores.

We then computed the Pearson correlations between the drift rate and boundary parameters of the LBA model and the questionnaire data (as summarized in Table 1). This analysis yielded one notable correlation: a negative correlation (*r*(178) = −0.258), BF > 50) between the counter-normative drift rate for normatively pleasant images (associated with evidence accumulation efficiency for unpleasant emotions) and the TEPS consummatory scale. The scatterplot of this correlation is presented in Figure 4. The remaining correlations were weak, with inconclusive Bayes Factors. The full set of correlation analysis is provided in the Supplementary Materials. In addition, three additional non-pre-registered analyses were conducted to further explore this result; as they did not clarify the pattern of findings, they are reported in the Supplementary Materials.

## 4. Discussion

In this study, we aimed to assess the convergent and discriminant validity of emotion-relevant parameters as derived using the LBA model. Unlike in most of its applications, the LBA model was fit to self-reports of (un)pleasant emotional experiences. To this end, participants completed a task in which they viewed emotion-evoking images from two distinct normative categories, slightly positive and slightly negative, and indicated whether the image made them feel pleasant. Their responses and reaction times were jointly modeled by the LBA model, which, in turn, generated latent variables (parameters) that describe the emotional experience report process. We focused on two key parameters: the drift rate, which reflects the speed of evidence accumulation (emotion accumulation efficiency), and the boundary, which reflects the level of decision caution. The drift rate was estimated separately for each combination of stimulus valence and the normative status of the response (normative vs. counter-normative). The boundary was estimated as a function of the response (pleasant vs. unpleasant). In addition to the emotion report tasks, participants completed five questionnaires: (1) the Positive and Negative Affect Schedule (PANAS; Watson et al., 1988), which assesses current positive and negative affective states; (2) the Mood and Anxiety Symptoms Questionnaire (MASQ; Clark & Watson, 1991), measuring symptoms of anxiety and depression over the past week; (3) the Temporal Experience of Pleasure Scale (TEPS; Gard et al., 2006), assessing general levels of anticipatory and consummatory pleasure; (4) the Center for Epidemiologic Studies Depression Scale—Revised (CESD-R 10; Andresen et al., 1994), evaluating depressive symptoms over the past week; and (5) the Snaith–Hamilton Pleasure Scale (SHAPS; Snaith et al., 1995), which assesses levels of anhedonia over the past week. We then examined the correlations between the scales derived from the questionnaire data and the LBA parameters of focus.

Of all correlations tested, only one reached substantial evidence levels (BF > 10), a negative correlation between consummatory pleasure and the counter-normative drift rate for normatively pleasant images (*r*(178) = −0.258), indicating that those participants reporting higher consummatory pleasure had lower unpleasant emotional accumulation efficiency toward a normatively pleasant stimulus. This correlation is consistent in its direction with our hypotheses. This result offers some initial support for the construct validity of LBA parameters in modeling affective decision-making.

Since the TEPS consummatory scale was the only questionnaire whose scales show significant correlation with an LBA parameter (specifically, a parameter that is estimated during the viewing of normatively pleasant emotional images), we tried to examine what makes the TEPS consummatory scale an exception. The anticipatory scale measures expected emotional responses to future experiences (“When ordering something off the menu, I imagine how good it will taste”), whereas the consummatory scale assesses immediate responses to present experiences (“A hot cup of coffee or tea on a cold morning is very satisfying to me”). Both scales ask about typical experiences, but they differ in temporal focus: expectation versus in-the-moment enjoyment. In the emotion task, participants were asked to report how pleasant each image made them feel now, capturing their immediate, in-the-moment emotional response to specific stimuli. This conceptual alignment (i.e., in-the-moment) may explain why the TEPS consummatory scale uniquely correlates with the LBA parameters, while the TEPS anticipatory scale does not.

Another key distinction between the TEPS consummatory scale and the other scales (with the exception of the TEPS anticipatory scale) is that the other scales assess retrospective evaluations of emotional experiences, such as emotions felt over the past week or in imagined scenarios. The PANAS is an exception as it asks participants to rate their current emotional state. However, the PANAS ratings are not tied to any specific stimulus and instead reflect the individual’s general feeling at the time of assessment. In contrast, the TEPS consummatory scale uniquely captures in-the-moment emotional responses to concrete stimuli, aligning it more closely with the emotion task.

The TEPS instructions further enhance its alignment with the emotion task. When participants have not personally experienced a specific situation described in the TEPS, they are instructed to imagine a similar one. This process of mental simulation may evoke momentary emotional experiences, resembling the immediate affective responses elicited by the emotion task, as research suggests that recalling past emotional events can elicit genuine emotional responses (Mulligan & Scherer, 2012).

More broadly, the present findings suggest that while the emotion task and self-report questionnaires measure a common underlying construct—emotions—they likely capture distinct facets of it, reflecting the multifaceted nature of emotions. This results in the lack of correlation between the self-reported measures and the behavioral task, a discrepancy that is not uncommon in studies comparing behavioral tasks with questionnaire-based assessments (Rizvi et al., 2016). As such, the lack of correlation between the LBA metrics from the emotion tasks and the measures other than the TEPS consummatory scale may make some theoretical sense. Specifically, these questionnaires do not directly assess the intensity or quality of emotional experiences themselves but rather related, yet conceptually distinct, facets such as anhedonia and depressive symptoms. As such, the lack of association may support the discriminant validity of the LBA parameters as a measure of emotional experience, suggesting that they capture core emotional responses rather than broader affective or clinical states. From this perspective, some of the null findings, although contrary to our original hypotheses, may in fact strengthen the central aim of the present work, namely, to evaluate whether the model captures emotional experience in a valid and specific manner. The fact that the only observed association involved a construct directly indexing emotional experience provides initial and modest support for convergent validity, whereas the absence of associations with related constructs points to relatively strong discriminant validity. We emphasize that this interpretation is post hoc and should be considered as such. To further clarify this post hoc interpretation, we note that among the questionnaires included, only the TEPS consummatory scale and the PANAS are conceptually aligned with convergent validity, given their focus on present-moment affective experience. Nevertheless, neither measure maps perfectly onto the construct assessed by our task. Specifically, the PANAS reflects current affects that are not tied to specific eliciting stimuli, and, given that we averaged PANAS scores across three sessions, it functioned here more as a trait-like indicator than as a state measure. Similarly, while the TEPS consummatory scale focuses on affective responses to specific stimuli, it asks about typical experiences rather than real online reactions to a stimulus. Still, given that both questionnaires capture an aspect of experiential affect that is related to the construct targeted by our task, they represent the best available approximations for construct validity. In contrast, the remaining questionnaires, including the MASQ Anxiety scale (anxiety), the MASQ Anhedonia scale (anhedonic depressive symptoms), the TEPS Anticipatory Pleasure scale (anticipated pleasure for future or imagined situations), the CESD-R 10 (severity of depressive symptoms), and the SHAPS (anhedonia), are better suited for evaluating discriminant validity, as they assess constructs that are theoretically related to, yet distinct from, online, stimulus-elicited emotional experience.

Several limitations of the current study may help explain some of the findings. First, the emotional stimuli used in our task were relatively mild in nature. In contrast, the PANAS includes items reflecting high-intensity affective states. It is possible that the use of more emotionally intense stimuli would have elicited stronger affective responses, potentially resulting in stronger correlations between the task-based model parameters and PANAS scores. Notably, there is a version of the PANAS that includes lower-arousal emotional items (Watson & Clark, 1994), which may have been a better match for the current stimulus set. Second, our study involved a non-clinical sample, which likely exhibited a restricted range in symptom severity and emotional variability. This restriction may have attenuated potential associations between self-report measures and model-based parameters, limiting the ability to detect robust effects. Third, while we acknowledge the limitations of self-reports in capturing emotional experiences (e.g., biases, retrospection), we relied here on self-reports to validate our computational modeling approach. This introduces an important theoretical tension: how can we meaningfully assess the validity of a model using tools we criticize? While this issue is not unique to our study, it remains an important consideration when interpreting the results. Finally, one practical limitation relates to the number of stimuli analyzed. Of the original 150 images, 35 were excluded based on our pre-registered criteria. This exclusion likely stems from the fact that image norms were derived from a Polish sample, whereas our participants were based in the United States. Cultural differences in emotional interpretation may have contributed to the mismatch, resulting in a substantial drop in usable trials. Therefore, it is possible that a greater number of trials would have yielded more stable parameter estimates and potentially uncovered additional patterns in the data.

Beyond limitations that may account for some of the observed results, several more general limitations should be acknowledged. Despite its advantages, the present approach, like any method, has inherent constraints. One such limitation concerns the response format, which required participants to indicate whether an image felt pleasant or not pleasant. Formally, this format does not explicitly distinguish between unpleasant and neutral experiences and therefore allows, in principle, for a pleasant–neutral interpretation. However, we consider this interpretation less likely for several reasons. Linguistically, describing an experience as not pleasant typically denotes the negation of pleasantness and is commonly (though not exclusively) used to refer to an unpleasant experience rather than a neutral one. In addition, the task instructions included an example emphasizing that participants should report pleasant when their subjective experience was pleasant, even if the stimulus was normatively negative, which framed the alternative response as reflecting an unpleasant experience. Finally, the pattern of results observed in the present study is more coherently interpreted if the not pleasant response is taken to reflect unpleasant emotional experience rather than neutrality. Nevertheless, we acknowledge this potential ambiguity as a limitation. An additional limitation concerns the forced choice between pleasant and unpleasant, which further constrains the range of reported experiences by focusing exclusively on valence. Nevertheless, we view this as a necessary starting point that must be established before moving to more complex choice structures and the modeling of discrete emotions such as joy or anger.

## Figures and Tables

**Table 1:** Summary of all hypotheses and exploratory analyses. Cor represents Pearson correlation.

Questionnaire	Scale	Pleasant Emotional Accumulation Efficiency Parameters	Unpleasant Emotional Accumulation Efficiency Parameters	Boundary Parameter for Pleasant Reactions	Boundary Parameter for Unpleasant Reactions
PANAS	Current positive emotional experience	Cor > 0	Cor < 0	Cor > 0	Cor > 0
	Current negative emotional experience	Cor < 0	Cor > 0	Cor > 0	Cor > 0
MASQ	Anxiety	Exploratory	Exploratory	Exploratory	Cor < 0
	Anhedonia	Cor < 0	Cor > 0	Exploratory	Cor < 0
TEPS	Anticipatory pleasure	Exploratory	Exploratory	Exploratory	Exploratory
	Consummatory pleasure	Cor > 0	Cor < 0	Cor > 0	Cor > 0
CESD-R 10	Severity of depressive symptoms	Exploratory	Exploratory	Exploratory	Exploratory
SHAPS	Anhedonia	Cor < 0	Cor > 0	Cor < 0	Cor < 0

**Table 2:** Sample sizes and descriptive statistics for each questionnaire.

Questionnaire	Scale	Number of Participants	Questionnaire Mean Value	Questionnaire Standard Deviation Value
PANAS	Current positive emotional experience	181	24.47	7.2
	Current negative emotional experience	181	15.89	5.46
MASQ	Anxiety	184	22.79	8.09
	Anhedonia	184	59.83	14.5
TEPS	Anticipatory pleasure	183	44.94	6.33
	Consummatory pleasure	183	37.05	6.26
CESD-R 10	Severity of depressive symptoms	184	8.98	5.61
SHAPS	Anhedonia	183	1.71	2.11

## Data Availability

All code and Data are available here (https://osf.io/c9dx2/overview?view_only=2b6dfc9375374b329a73cb62aaac2321, accessed on 11 January 2026).

## Notes

1 Due to a misinterpretation of the scale direction, where higher values represent greater anhedonia rather than increased pleasure, the hypotheses stated in the pre-registration for the SHAPS questionnaire were formulated in the opposite direction.

2 In the pre-registration, we specified that outlier detection would be conducted using these plots. However, we applied a more stringent criterion, identifying participants as outliers if their values deviated by at least three standard deviations from the sample mean. Upon further consideration, we determined that visual inspection alone was sufficient for identifying outliers. This decision was made prior to the correlations analysis.

## Supplementary Materials

The following supporting information can be downloaded at: https://www.mdpi.com/article/10.3390/jintelligence14020019/s1. Figure S1: Density plot for each scale; Figure S2: Scatterplots of notable correlations between drift rate parameters and questionnaire scores; Figure S3: Pearson correlation between all scale (questionnaires) combinations; Table S1; Table S2.
