# Supplementary material for: Don’t Tell Us How Strong It Feels! Converging and Discriminant Validity of an Indirect Measure of Emotional Evidence Accumulation Efficiency
Source: J Intell. 2026 Jan 31;14(2):19. doi: 10.3390/jintelligence14020019 (PMC12941456; doi:10.3390/jintelligence14020019)
Supplement: Supplementary file 1 [file jintelligence-14-00019-s001.zip › jintelligence-3966444-supplementary.pdf]

Don't Tell Us How Strong It Feels! Converging and Discriminant Validity of an Indirect Measure of Emotional Evidence Accumulation Efficiency

To identify outliers, we plotted the statistics for each questionnaire. Those plots are provided in Figure S1.

Pre-registered Main model

For the Pre-registered Main models, the PSRF values for all participants were below 1.1, indicating satisfactory chain convergence. Additionally, its summed DIC values were 36,454. Given that a lower DIC value reflects a better model fit, this result provides evidence in favor of the Pre-registered Main model over the Null model. Furthermore, for 63.78% of the sample, the DIC was lower in the Pre-registered Main model compared to the Null model. Collectively, these findings support the Pre-registered Main model over the Null model. Additionally, RMSEA values fell below 0.08, indicating a good model fit (RMSEA= .0477, 95% CI [.0472, .0483]). The mean posteriors of the Pre-registered Main model were as follows: A=1.398, b positive valence = 1.352, b negative valence = 1.44, t0 = 0.202, v normative positive valence = 1.789, v normative negative valence = 2.34, v counter-normative positive valence = 0.59, and v counter-normative negative valence = 0.286, sv= 0.943.

We then computed the Pearson correlations between the drift rate and boundary parameters of the LBA model and the questionnaire data. This analysis yielded two notable correlations: a positive correlation (*r*(178) =.249, BF >50) between the normative drift rate for normatively pleasant images (associated with evidence accumulation efficiency for pleasant emotions) and the TEPS Consummatory scale, and a negative correlation (*r*(178) = -.274, BF >100) between the counter-normative drift rate for normatively pleasant images (associated with evidence accumulation efficiency for unpleasant emotions) and the TEPS Consummatory scale. The scatterplots of these two correlations are presented in Figure S1. The remaining correlations were weak, with inconclusive Bayes factors. The full set of correlation analysis is provided in the Supplementary Materials. Beyond these two correlations, we also explored additional

correlations that produced inconclusive findings. These results are presented in Table S1.

Corrected superior model

Aside from the correlation reported in the main text, we tested additional correlations. All correlation results are shown in Table S2.

Additional non-pre-registered analyses

Following these findings, we considered what distinguishes the TEPS Consummatory scale from the other questionnaires included in the study. We found that one of the things that distinguishes it is the inclusion of items that describe physical sensory experiences (e.g., “I love the sound of rain on the windows when I'm lying in my warm bed”). We hypothesized that reporting current emotional experiences, as required in the emotion task, may involve a process of introspection into physical sensations. To empirically evaluate this hypothesis, we identified and extracted all items from the other questionnaires that similarly referenced physical sensations. These items were drawn from three scales: MASQ Anxiety, MASQ Anhedonia, and SHAPS. We then constructed a new scale, "the physical sensations scale", which was scored by summing the relevant item responses, accounting for the questionnaire’s scaling differences. The resulting scale was based on data from 174 participants, and it yielded a mean score of 164.2 and a standard deviation of 34.51. Next, we examined Pearson correlations between the new physical sensations scale and each of the four drift rate parameters from the LBA model. However, none of the correlations were noteworthy. All correlation results are summarized in Tables 1 and 2.

We used items from three scales (MASQ Anxiety, MASQ Anhedonia, and SHAPS) to build the new Sensation scale. Those items were as follows:

MASQ Anxiety

″Hands were shaky", "Was short of breath", "Felt faint", "Had hot or cold spells", "Hands were cold or sweaty", "Was trembling or shaking", "Had trouble swallowing", "Felt dizzy or lightheaded", "Had pain in my chest", "Felt like I was choking", "Muscles twitched or trembled", "Had a very dry mouth", "Heart was racing or pounding", and "Felt numbness or tingling in my body".

MASQ Anhedonia

"Had to urinate frequently".

SHAPS

"I would be able to enjoy my favourite meal", "I would enjoy a warm bath or refreshing shower", "I would find pleasure in the scent of flowers or the smell of a fresh sea breeze or

freshly baked bread", "I would enjoy a cup of tea or coffee or my favourite drink". "I would find pleasure in small things, e.g. bright sunny day, a telephone call from a

friend", and "I would be able to enjoy a beautiful landscape or view".

To ensure comparability across questionnaires that use different response scales, we rescaled each participant's responses to a uniform metric. Specifically, SHAPS responses (originally on a 1-4 scale) were multiplied by 5, and MASQ responses (originally on a 1-5 scale) were multiplied by 4. The rescaled items were then summed to construct the new Sensation scale.

The two additional non-pre-registered analyses, which were mentioned in the main text, were conducted. First, we explored whether the relationships between questionnaire scores and

LBA model parameters differed by gender. To test this, for each comparison we fitted two regression models in which a questionnaire score served as the dependent variable. In one model, an LBA parameter and gender were (additive) predictors. In the other model, the interaction between gender and the LBA parameter was also included. We then compared these models using Bayesian model comparison. However, all comparisons yielded inconclusive results.

In addition, we examined the correlations among the self-report scales. The results of this analysis are presented in Figure S3.

Figure S1

Density plot for each scale

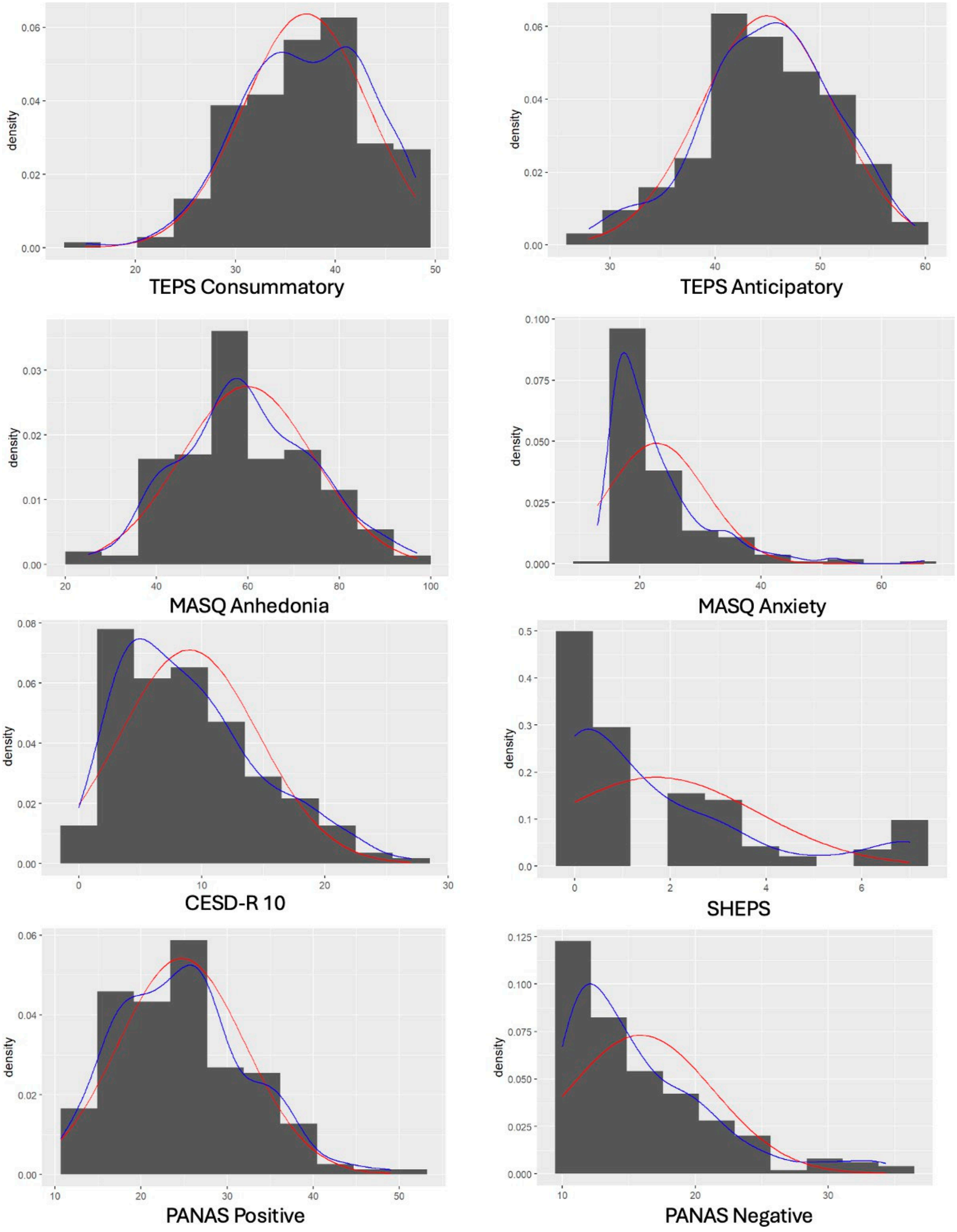

Figure S2

Scatterplots of notable correlations between drift rate parameters and questionnaire scores

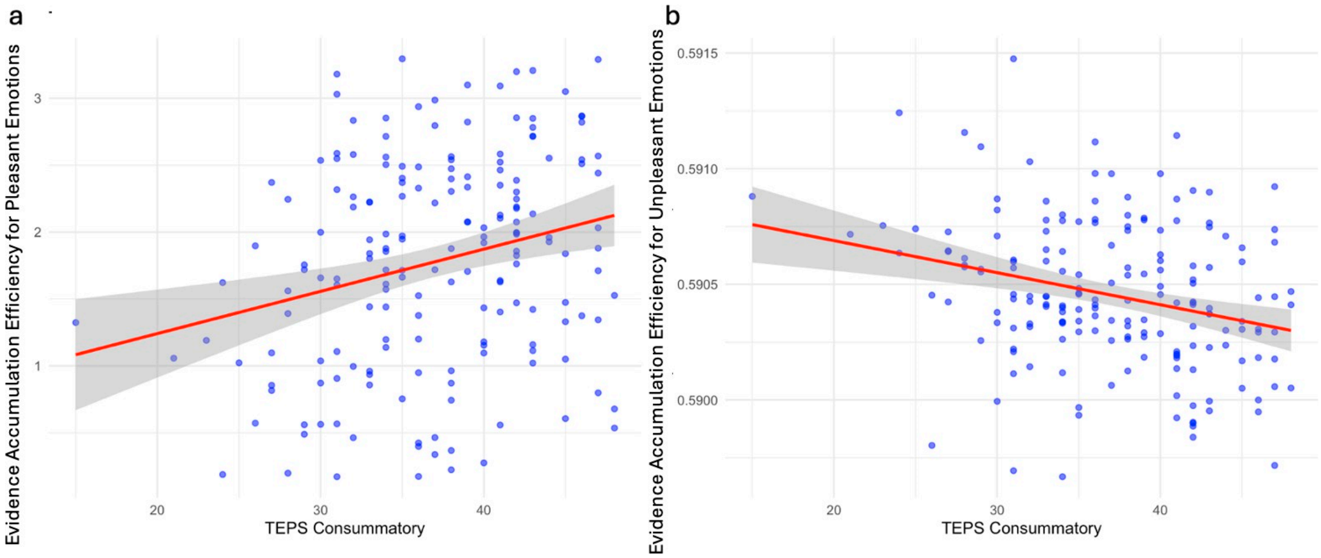

(a) Scatterplot of the correlation between the normative drift rate for normatively pleasant images (associated with evidence accumulation efficiency for pleasant emotions) and the TEPS Consummatory questionnaire. The Y-axis represents evidence accumulation efficiency for pleasant emotions, as modelled using the normative drift rate for normatively pleasant images. (b) Scatterplot of the correlation between the normative drift rate for counter-normatively pleasant images (associated with evidence accumulation efficiency for unpleasant emotions) and the TEPS Consummatory questionnaire. The Y-axis represents evidence accumulation efficiency for unpleasant emotions, as modelled using counter-normative drift rate for normatively pleasant images. In both panels, the TEPS Consummatory scale is represented on the X-axis.

Figure S3

Pearson correlation between all scale (questionnaires) combinations

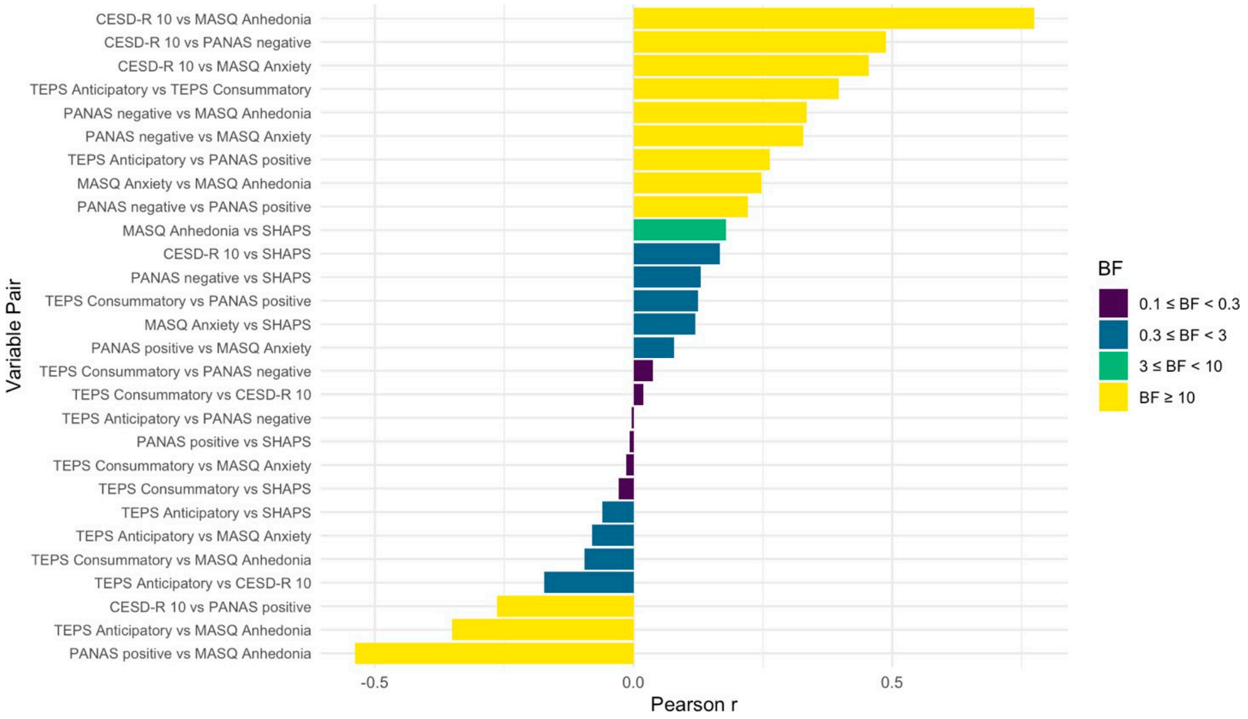

Bar plot displaying the Pearson correlation coefficients between all possible pairs of questionnaire scales. Each bar represents a unique pairwise combination, listed on the Y-axis. The X-axis indicates the strength and direction of the correlation. Bar colors represent the corresponding BF, providing an estimate of the strength of evidence for each correlation.

Table S1

Questionnaire	Scale	Evidence accumulation efficiency for pleasant emotions parameters		Evidence accumulation efficiency for unpleasant emotions parameters		Boundary normatively pleasant stimuli	Boundary normatively unpleasant stimuli
		Normative accumulator for normatively pleasant stimuli	Counter-normative accumulator for normatively unpleasant stimuli	Normative accumulator for normatively unpleasant stimuli	Counter-normative accumulator for normatively pleasant stimuli		
PANAS	Current positive emotional experience	*r*= .100, *BF*= .535	*r*= .067, *BF*= .334	*r*= -.095, *BF*= .491	*r*= -.117, *BF=.738*	*r*= -.004, *BF*=.229	*r*= -.034, *BF*= .253
	Current negative emotional experience	*r*= -.023, *BF*=.24	*r*= .000, *BF*=.229	*r*= -.010, *BF*= .231	*r*= .026, *BF*= .242	*r*= -.049, *BF*= .281	*r*= .043, *BF*= .268
MASQ	Anxiety	*r*= .039, *BF*= 0.26	*r*= .153, *BF*= 1.70	*r*= .010, *BF*= .231	*r*= .058, *BF*=.306	*r*= -.013, *BF*=.232	*r*= -.057, *BF*=.031
	Anhedonia	*r*= .080, *BF*=.395	*r*= -.038, *BF*= .26	*r*= .035, *BF*= .254	*r*= .078, *BF*= .382	*r*= -.036, *BF*= .256	*r*= .094, *BF*=.488
TEPS	Anticipatory pleasure	*r*= .113, *BF*= .687	*r*= .118, *BF*= .755	*r*= -.067, *BF*= .336	*r*= .120, *BF*= .509	*r*= .065, *BF*= .329	*r*= -.120, *BF*= .509
	Consummatory pleasure	*r*= .249, *BF*= 50.570	*r*= .148, *BF*=1.548	*r*= .136, *BF*= 1.110	*r*= -.274, *BF*= 169.517	*r*= .202, *BF*= 7.739	*r*= -.097, *BF*= .509
CESD-R 10	Severity of depressive symptoms	*r*= .045, *BF*= .272	*r*= .036, *BF*= .256	*r*= .019, *BF*= .236	*r*= .022, *BF*= .239	*r*= -.029, *BF*= .246	*r*= .002, *BF*= .299
SHAPS	Anhedonia	*r*= -.015, *BF*= .233	*r*= .102, *BF*= .544	*r*= -.037, *BF*= .257	*r*= -.017, *BF*= .235	*r*= -.038, *BF*= .260	*r*= .037, *BF*= .258

Combination	Sensations scale	*r*= -.208, *BF*= .492	*r*= .193, *BF*= 5.23	*r*= .036, *BF*= .259	*r*= .014, *BF*= .236	–	–

Table S2

Questionnaire	Scale	Evidence accumulation efficiency for pleasant emotions parameters		Evidence accumulation efficiency for unpleasant emotions parameters		Boundary pleasant reaction	Boundary unpleasant reaction
		Normative accumulator for normatively pleasant stimuli	Counter-normative accumulator for normatively unpleasant stimuli	Normative accumulator for normatively unpleasant stimuli	Counter-normative accumulator for normatively pleasant stimuli		
PANAS	Current positive emotional experience	*r*= .037, *BF*= .257	*r*= -.006, *BF*= .229	*r*= .047, *BF*= .277	*r*= .095, *BF*= .492	*r*= .045, *BF*= .273	*r*= -.081, *BF*= .402
	Current negative emotional experience	*r*= -.013 , *BF*= .232	*r*= -.032, *BF*= .25	*r*= -.017, *BF*= .235	*r*= .073, *BF*= .363	*r*= .003, *BF*= .229	*r*= .003, *BF*= .229
MASQ	Anxiety	*r*= .07, *BF*= .349	*r*= .177, *BF*= 3.379	*r*= .000, *BF*= .22	*r*= .07, *BF*= .348	*r*= .000, *BF*= .22	*r*= -.069, *BF*= .344
	Anhedonia	*r*= .002, *BF*= .22	*r*= -.014 , *BF*= .233	*r*= -.027, *BF*= .243	*r*= .01, *BF*= .538	*r*= -.009, *BF*= .23	*r*= .069, *BF*= .342
TEPS	Anticipatory pleasure	*r*= .024, *BF*= .241	*r*= .097, *BF*= .512	*r*= -.007, *BF*= .23	*r*= -.132, *BF*= 1.023	*r*= .042 , *BF*= .265	*r*= -.106, *BF*= 1.846
	Consummatory pleasure	*r*= .106, *BF*= .599	*r*= .084, *BF*= .418	*r*= .033, *BF*= .251	*r*= -.258, *BF*= 76.518	*r*= .199, *BF*= 6.919	*r*= -.156, *BF*= 1.846
CESD-R 10	Severity of depressive symptoms	*r*= .081, *BF*= .4	*r*= .01 , *BF*= .231	*r*= .026 , *BF*= .242	*r*= .061, *BF*= .316	*r*= .004, *BF*= .229	*r*= -.064, *BF*= .325
SHAPS	Anhedonia	*r*= -.022, *BF*= .238	*r*= .088, *BF*= .441	*r*= -.064, *BF*= .323	*r*= .017, *BF*= .234	*r*= .01, *BF*= .231	*r*= -.044, *BF*= .27

Combination	Sensations scale	*r*= .1, *BF*= .53	*r*= .182, *BF*= 3.598	*r*= .0511, *BF*= .287	*r*= .042, *BF*= .268	-	-
